# Over-Expression of the Mycobacterial Trehalose-Phosphate Phosphatase OtsB2 Results in a Defect in Macrophage Phagocytosis Associated with Increased Mycobacterial-Macrophage Adhesion

**DOI:** 10.3389/fmicb.2016.01754

**Published:** 2016-11-04

**Authors:** Hao Li, Mei Wu, Yan Shi, Babak Javid

**Affiliations:** ^1^Collaborative Innovation Centre for the Diagnosis and Treatment of Infectious Diseases, School of Medicine, Tsinghua UniversityBeijing, China; ^2^Tsinghua Immunology Institute, School of Medicine, Tsinghua UniversityBeijing, China

**Keywords:** *Mycobacterium bovis*-BCG, OtsB2, trehalose, macrophage, phagocytosis, adhesion

## Abstract

Trehalose-6-phosphate phosphatase (OtsB2) is involved in the OtsAB trehalose synthesis pathway to produce free trehalose and is strictly essential for mycobacterial growth. We wished to determine the effects of OtsB2 expression on mycobacterial phenotypes such as growth, phagocytosis and survival in macrophages. *Mycobacterium bovis*-bacillus calmette-guerin (BCG) over-expressing OtsB2 were able to better survive in stationary phase. Over-expression of OtsB2 led to a decrease in phagocytosis but not survival in THP-1 macrophage-like cells, and this was not due to a decrease in general macrophage phagocytic activity. Surprisingly, when we investigated macrophage–mycobacterial interactions by flow cytometry and atomic force microscopy, we discovered that BCG over-expressing OtsB2 have stronger binding to THP-1 cells than wild-type BCG. These results suggest that altering OtsB2 expression has implications for mycobacterial host–pathogen interactions. Macrophage–mycobacteria phagocytic interactions are complex and merit further study.

## Introduction

*Mycobacterium tuberculosis* is the causative agent of tuberculosis (TB) and is responsible for more than 1.5 million deaths and 9 million cases of active TB per year. As the only licensed vaccine for tuberculosis, *M. bovis*-bacillus calmette-guerin (BCG) is one of the most widely used vaccines in the world, but it has limited efficacy (Mangtani et al., 2014). A better understanding of how BCG interacts with host immunity may allow rational design of improved tuberculosis vaccines.

Trehalose, a non-reducing disaccharide of glucose, is a key metabolic intermediate in bacterial and eukaryotic physiology – it is made by bacteria, plants, fungi and insects, but not mammals (Tzvetkov et al., 2003; Iturriaga et al., 2009). Trehalose is a key component of trehalose dimycolate (TDM, cord factor), a major mycobacterial virulence factor (Kalscheuer and Koliwer-Brandl, 2014) and other cell-wall associated glycolipids (Kaur et al., 2009). Although mycobacteria have three described pathways for trehalose biosynthesis (De Smet et al., 2000; Woodruff et al., 2004), it is thought that the OtsAB pathway is the dominant pathway (Murphy et al., 2005), and is required for growth of *M. tuberculosis in vitro* and virulence (Murphy et al., 2005). The OtsAB pathway involves the condensation of glucose-6-phosphate with UDP-glucose to form trehalose-6-phosphate by trehalose-6-phosphate synthase (OtsA), and the subsequent generation of trehalose by trehalose-phosphate-phosphatase (TPP – OtsB; Kalscheuer and Koliwer-Brandl, 2014). The *M. tuberculosis* genome codes for two homologs of *otsB. otsB1* (Rv2006) and *otsB2* (Rv3372), but only the latter possesses true TPP activity and is essential for mycobacterial growth (Murphy et al., 2005).

Unlike some intra-cellular pathogens such as *Listeria monocytogenes* and *Salmonella enterica* subsp. *enterica* that actively induce phagocytosis of host cells (Asrat et al., 2014), mycobacteria are unable to induce phagocytosis, and therefore rely on the phagocytic activity of the host cell (He et al., 2012; Stanley and Cox, 2013). The initial step in non-opsonic phagocytosis of mycobacteria involves passive engagement of host plasma-membrane phagocytic receptors with their corresponding mycobacterial ligand, following which ingestion of the mycobacterial particle occurs (Schafer et al., 2009). Numerous, redundant receptors mediating phagocytosis exist, making study of the contribution of individual receptors difficult (Philips et al., 2005; Schafer et al., 2009; Killick et al., 2013). The reason for this redundancy is not clear – it may be that different receptors traffic the phagocytosed mycobacteria to alternate intra-cellular compartments – but selective inhibition of a subset of these receptors surprisingly did not influence mycobacterial survival (Zimmerli et al., 1996). Mycobacterial factors mediating phagocytosis are less well understood. The trehalose derived cord factor, trehalose 6,6′-dimycolate (TDM) can be recognized by multiple receptors such as Mincle, Dectin-3, MARCO and TLR-2 (Bowdish et al., 2009; Ishikawa et al., 2009; Zhao et al., 2014), underlying the importance of trehalose as a pathogen-associated molecular pattern.

We decided to investigate the effects of OtsB2 over-expression in BCG and found decreased phagocytosis by THP-1-derived macrophages of BCG expressing more OtsB2, and this was paradoxically associated with tighter binding to macrophages.

## Materials and Methods

### Bacterial Strains and Growth Conditions

*Mycobacterium bovis* BCG Pasteur 1173P2 strain was grown on Middlebrook 7H10 agar (Difco) plates supplemented with Oleic Acid-Albumin-Dextrose-Catalase (OADC) enrichment or in 7H9 medium supplemented with 10% OADC, 0.5% glycerol, and 0.05% Tween-80, in the presence of hygromycin (50 μg/ml) or kanamycin (25 μg/ml) for transformation selection and for growth of transformants, as indicated. Plates were incubated at 37°C for 3 weeks before counting of the colony forming units (CFU). Strains used for cloning was *Escherichia coli DH5*α (Tiangen Corp, China) that was grown in LB medium containing Hygromycin 150 μg/ml or kanamycin (50 μg/ml) at 37°C.

### Construction of an *M. bovis*-BCG Strain Over-expressing OtsB2

The OtsB2 gene was PCR-amplified from H37Rv chromosomal DNA using the primers PU1: 5′-CCTTAATTAA*GAAGGAGATATACAT*ATG **CATCACCATCACCATCAC**GTGCGCAAGTTGGGCC-3′ (Forward) and PU2: 5′-CCGATATCTTATCACGTTGCCCGCAGGGG-3′ (Reverse). A ribosomal binding site (italics) was included 5′ to the starting ATG start site, as well as a hexa-histidine affinity tag (bold) at the N-terminus of the protein. The OtsB2 gene was then subcloned into vector pUVtetOR, a tetracycline regulated mycobacterial shuttle plasmid using the restriction enzyme sites PacI (underlined, forward primer) and EcoRV (underlined, reverse primer).

The OtsB2 gene was subcloned into the plasmid pMV261 using the restriction enzyme sites MscI (forward, underlined) and HindIII (reverse, underline) and primers PM1:5′-CCTGGCCACATCACCATCACCATCACGTGCGCAAGTTGG-3′ (Forward) and PM2: 5′-CCAAGCTTTTATCACGTTGCCCGCAGGGG-3′ (Reverse). After constructing the recombinant plasmids, the plasmid was transformed into competent wild-type (WT) *M. bovis*-BCG by electroporation using standard settings of the electroporator. Recombinant strains were selected on Middlebrook 7H10 agar containing 50 μg/ml hygromycin or 25 μg/ml kanamycin as appropriate and after 3 weeks of incubation at 37°C then grown in enriched 7H9 medium.

### Western Blot Analysis

Bacillus calmette-guerin (WT or recombinant) was lysed by bead-beating. Lysate quantity was normalized using Bradford assay and equivalent amounts loaded onto a 12% polyacrylamide gel following addition of SDS loading buffer. After electrophoresis, proteins were transferred to a PVDF membrane by semi-dry transfer and blotted using standard procedures with monoclonal antibody to hexahistidine (2A11 Abcam) and secondary antibody using standard protocols. Blots were visualized by ECL reagent (Thermo) and a Biorad image analyser.

### Growth Characteristics of pTet-OtsB2 *In vitro*

The WT BCG and BCG-OtsB2 strains were grown in 7H9 medium until stationary phase and then diluted to OD_600_ 0.1 on Day 1. Growth was monitored by measuring OD_600_ or by plating for CFU.

### Trehalose Determination by Anthrone Method

The WT BCG and BCG-OtsB2 strains were grown to early log phase and stationary phase. The bacteria were lysed by beadbeater for 3 min with 0.1 mm beads (Biospec,USA). The trehalose-containing fraction of the lysate was extracted using 0.5 M trichloroacetic acid and anthrone was used to react with the BCG trehalose extracts and the products were measured at 620 nm, according to the protocol by Brin (1966). A linear relationship was obtained between trehalose concentration and absorbance in a standard curve, which was used to calibrate trehalose content of bacterial cells.

### THP-1 Cells Differentiation and Bacterial Infection

Human monocyte cell-line THP-1 cells were cultured in RPMI-1640 supplemented with 10% (v/v) FBS (THP-1 culture medium) and 1% (v/v) Penicillin/Streptomycin (except just prior to infection assays) at 37°C in 5% CO_2_ (v/v) incubator. The suspended THP-1 cells were harvested by centrifuging at 1000 rpm for 5 min and plated in six well plates containing cover-slips or not as required. Then 100 nM PMA was added to the medium and the cells were differentiated into macrophages after 24 h in a 37°C/5% CO_2_ incubator.

Bacterial clumps were discarded by centrifugation at 300 × *g* for 2 min and then the supernatant containing less clumped bacteria was harvested by centrifugation at 4000 rpm for 10 min. The pellets were then washed by PBS three times and then re-suspended in THP-1 culture medium (without antibiotics). Single-cell suspensions of the bacterial culture were obtained by multiple passage through a 25guage needle and then the suspension was filtered through 5 μm filters. Approximate cell density was calculated by measurement of OD_600._ The activated THP-1 cells were infected with BCG at different MOI (see Figure legends) for different time points at 37°C/5% CO_2_ condition.

### RAW264.7 Macrophage Culture

Murine RAW264.7 cells were cultured in DMEM medium separately supplemented with 10% (v/v) FBS and 1% (v/v) Penicillin/Streptomycin (except just prior to infection assays) at 37°C in 5% CO_2_ (v/v) incubator. The RAW264.7 cells were harvested by treating with 0.05% trypsin and then washed by PBS and plated with culture medium in 6 well plates.

### Colony Forming Unit (CFU) Assay

After 2 or 24 h infection, the infected macrophages were washed three times with PBS and then 500 μl of lysis buffer (sterile water 0.1% triton-100) was added to the wells at 37°C for 10 min. The resulting lysate (with intact bacteria) were diluted and plated on 7H10 plates for enumeration. The plates were placed in a 37°C incubator for 3∼4 weeks and then the colonies were counted. Each test was done three times in independent experiments, and the number of CFU recovered per well (mean ± SD) was determined.

### Quantification of Phagocytosis by Confocal Microscopy

After 24 h infection, the bacteria were washed three times with PBS. The cells were then fixed by 4% paraformaldehyde for 30 min at room temperature. After further rinsing by PBS, the cells were stained by Auramine O for 20 min at room temperature. The cells were then washed three times by PBS and ddH2O, slides were decolorized thoroughly with TB Decolorizer (BD) until the stain disappeared. After washing well in ddH2O, mycobacteria were counter-stained by potassium permanganate for 4∼5 min. After further rinsing, the coverslips were mounted on glass slides by ProLong^®^ Antifade Kit (Invitrogen, USA). The images were taken with a confocal laser scanning microscope (LSM780, Zeiss). Data are expressed as percent phagocytosis, calculated as the total number of cells with at least one bacteria relative to the total number of cells counted.

### Phagocytosis and Cell-Adhesion Assay by Flow Cytometry

FITC dye was dissolved in 0.1 M carbonate/bicarbonate buffer at the final concentration of 1 mg/ml. The BCG-WT and BCG-OtsB2 were collected by centrifuge at 4000 rpm for 10 min. The bacteria were stained by 1mg/ml FITC solution for 2 h on the shaker at room temperature. After staining, the bacteria were washed three times by PBS and the bacteria then passaged multiple times through a 25 gauge needle and then the resulting suspension was filtered by 5 μm filters to obtain a single-cell suspension of bacteria. Efficient staining (>99% FITC+) of the bacteria was verified by flow cytometry prior to further experiments. This single-cell suspension was used to infect activated THP-1 cells. For phagocytosis, the samples were put in the 37°C/5% CO_2_ (v/v) incubator. For the binding assay, the samples were put in the 4°C refrigerator in the dark. After infection, the cells were washed by 5% FCS/PBS three times and analyzed by a BD C6 flow cytometer. Data were analyzed using Flowjo software.

### Atomic Force Microscopy

Glass disk-adhered activated THP-1 cells were put onto the AFM stage. Five microliters of BCG-WT or BCG-OtsB2 bacterial suspension were added to the side of the glass disk and allowed to settle. The cantilever was slowly lowered to contact with the bacterium until the bacterium could adhere to the cantilever. The tip was then lowered to approach a single activated THP-1 cell until the bacterium could firmly contact with the THP-1 cells and the binding forces were collected.

### Beads Phagocytosis Assay

After the activated THP-1 cells were infected for 6 h at the MOI of 10:1 by BCG-WT or BCG-OtsB2 (both non-fluorescent), the cells were washed three times by PBS. Fluorescent particles (Spherotech, USA) were added into the wells (beads: cells ratio = 10:1). The cells were incubated with the beads at 37°C, 5% CO_2_ for 2 h. Non-internalized beads were then removed by washing with ice-cold PBS three times. Finally the macrophage cells were re-suspended in 500 μL of 5% FCS/PBS and then analyzed using the BD C6 cytometer. Forward and side scatter gates was set to exclude debris and to gate on macrophages. Data were analyzed using Flowjo software.

### Cell Surface Staining and Flow Cytometry

After the activated THP-1 cells were infected for 24 h at the MOI of 5:1, the cells were washed with the staining buffer (5% FBS/PBS). The infected macrophages were collected with 10 mM EDTA/PBS solution for 10 min at room temperature. The cell surface expression of CD80 (clone:2D10.4), CD86 (clone:IT2.2), MHC I (clone: 34-1-2S), MHC II (clone:M5/114.15.2), CD1a (clone:HI149),CD1b (clone: eBioSN13),CD1c (clone: L161), CD1d (clone:51.1), TLR2(clone:TL2.1), TLR4(clone:HTA125), and CD14(clone:61D3) markers were measured. Dectin-1 and Mincle markers were measured using mouse monoclonal antibodies raised and kindly gifted by the Xin Lin Lab, Tsinghua University School of Medicine. The primary antibodies for each marker were used for staining. The cells were stained for about 1 h at 4°C, and then the samples were washed by the staining buffer for three times and then the samples were analyzed by the BD C6. Forward and side scatter gate was set to exclude debris. Data were analyzed using Flowjo software.

### Statistical Analysis

Data are expressed as mean ± SD. Data were compared by two-tailed unpaired Student’s *t*-tests using the computer program Prism version 5.0 (GraphPad Software, USA). Differences were considered statistically significant when *p*-values < 0.05. *n* represents the number of experiments.

## Results

### BCG Over-Expressing OtsB2 Are Phagocytosed Less than Wild-Type BCG

We cloned the *M. tuberculosis otsB2* gene into the inducible episomal plasmid under the control of a tetracycline-inducible promoter, pTet (Ehrt et al., 2005). Induction by the tetracycline analog anhydrotetracycline (ATc-50 ng/ml) resulted in detectable expression of recombinant OtsB2 by western blot (**Supplementary Figure S1A**). Expression increased in stationary compared with log phase (**Supplementary Figure S1A**). We decided to test whether over-expression of OtsB2 in BCG affected bacterial growth. Although growth of the over-expressor strain and WT BCG were similar in lag and log phase, the over-expressor strain was able to grow to a greater cell density in stationary phase as measured by optical density (**Supplementary Figure S1B**) and CFU (not shown) and this was associated with an increase in cellular trehalose in later stationary phase as measured by the anthrone method (**Supplementary Figure S1C**).

**FIGURE 1:**
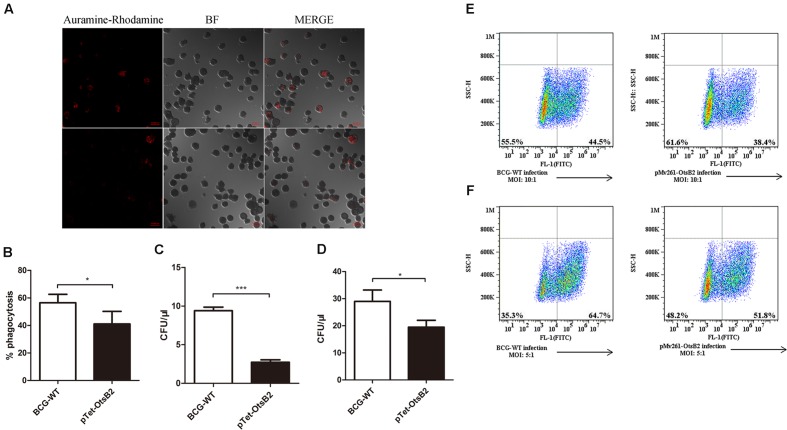
**BCG-OtsB2 is phagocytosed less than WT BCG by THP-1 macrophages.** **(A)** Confocal microscopy of Auramine O-stained WT BCG (upper panels) and BCG-OtsB2 (lower panels) following phagocytosis (MOI: 5:1) by activated THP-1 macrophages. **(B)** Numerical analysis of confocal data. **(C)** Survival by plated CFU of BCG and BCG-OtsB2 following phagocytosis by THP-1 macrophages for 2 h and **(D)** 24 h. MOI: 5:1. Phagocytosis analysis by flow cytometry of BCG vs BCG-OtsB2 at MOI 10:1 **(E)** and 5:1 **(F)**. ^∗^*p* < 0.05, ^∗∗∗^*p* < 0.001 by Student’s *t*-test. Results representative of at least three separate experiments.

Since over-expression of OtsB2 was associated with stationary phase survival, we wanted to determine whether it influenced infection of macrophages. We infected PMA-differentiated THP-1 cells, which behave similarly to human macrophages (Fleit and Kobasiuk, 1991; Daigneault et al., 2010) with BCG over-expressing OtsB2 (BCG-OtsB2) or WT BCG. By confocal microscopy, we discovered that THP-1 cells infected by BCG-OtsB2 had engulfed significantly fewer bacteria than those infected by WT BCG (**Figures 1A,B**). The number of surviving bacteria, as measured by plated CFU was proportional to the degree of phagocytosis at both early – 2 h (**Figure 1C**) and late – 24 h (**Figure 1D**) time points suggesting the defect was in phagocytosis, not survival. We could also detect the defect in phagocytosis by flow cytometry, and the phenotype was similar regardless of whether OtsB2 was over-expressed by induction of ATc or by constitutive over-expression by the episomal vector pMV261 (**Figures 1E,F**).

We wanted to determine whether the phagocytosis defect was due to a general ‘poisoning’ or damaging of the macrophages by BCG-OtsB2, which would render them generally defective in phagocytosis, or was specific to mycobacteria over-expressing OtsB2. We investigated whether infection of THP-1 macrophages by BCG-OtsB2 was associated with changes in cell-surface expression of a number of immune-related receptors including a number of receptors such as TLR2, TLR4, Mincle, Dectin-1 and CD-14 that have been implicated in phagocytosis of mycobacteria (Peterson et al., 1995; Ernst, 1998; Nicolle et al., 2004). Although there was no difference in cell-surface expression of MHC class I, class II, CD80, CD86 and CD49c, there was up-regulation of the alternative antigen presentation receptor CD1d when macrophages were infected by BCG-OtsB2, but not other CD1 types (**Supplementary Figure S2**), the significance of which was not clear. Although mycobacterial infection led to upregulation of TLR2, TLR4, and CD14, there was no difference in expression of any phagocytic receptors between macrophages infected by WT BCG and BCG-OtsB2 (**Supplementary Figure S3**).

Given the minor change in plasma-membrane phenotype of BCG-OtsB2 infected macrophages compared with those infected with WT BCG, to determine whether BCG-OtsB2 induced a general or specific phagocytosis defect, activated THP-1 cells were first infected by either WT BCG or BCG-OtsB2, then fed fluorescently labeled latex beads – there was no significant difference in the degree of phagocytosis of the latex beads between the two groups (**Supplementary Figure S4**), suggesting that rather than a general phagocytosis defect, THP-1 macrophages were specifically less able to phagocytose BCG over-expressing OtsB2.

### BCG Over-Expressing OtsB2 Mediates Strong Binding to THP-1 Macrophages

**FIGURE 2:**
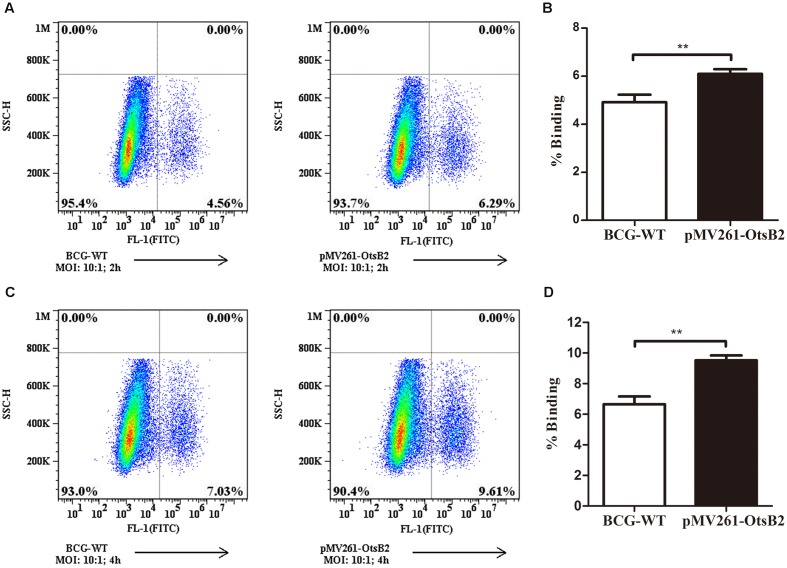
**BCG-OtsB2 has greater binding to THP-1 macrophages than WT BCG.** Binding analysis of BCG-OtsB2 and BCG to THP-1 macrophages at 4°C for 2 h **(A,B)** and 4 h **(C,D)**. ^∗∗^*p* < 0.01 by Student’s *t*-test. Results representative of at least three separate experiments.

We wondered whether mycobacteria over-expressing OtsB2 may have altered recognition by host macrophages, and hence decreased binding to the cell, mediating the decreased phagocytosis. At 4°C, macrophages are able to bind bacteria or other particulate matter, which is generally a passive process, but are unable to phagocytose the cells (Stokes et al., 2004), which is an active process. Therefore we determined the relative binding of WT BCG and BCG-OtsB2 to THP-1 macrophages at 4°C (**Figure 2**). Surprisingly, at both 2 and 4 h at cold temperatures, BCG-OtsB2 bound more to macrophages than WT BCG as defined by flow cytometry (**Figure 2**). To ensure that our observations were not limited to the interactions of BCG-OtsB2 and differentiated THP-1 cells, we repeated assays for both phagocytosis at 37°C and cell-binding only at 4°C with the RAW264.7 mouse macrophage cell-line. As can be seen from **Supplementary Figures S5A,B**, BCG-OtsB2 cells underwent less phagocytosis than WT BCG at 24 h, but paradoxically had stronger binding to the RAW macrophages when fed to the cells at 4°C when phagocytosis would be inhibited (**Supplementary Figures S5C,D**).

**FIGURE 3:**
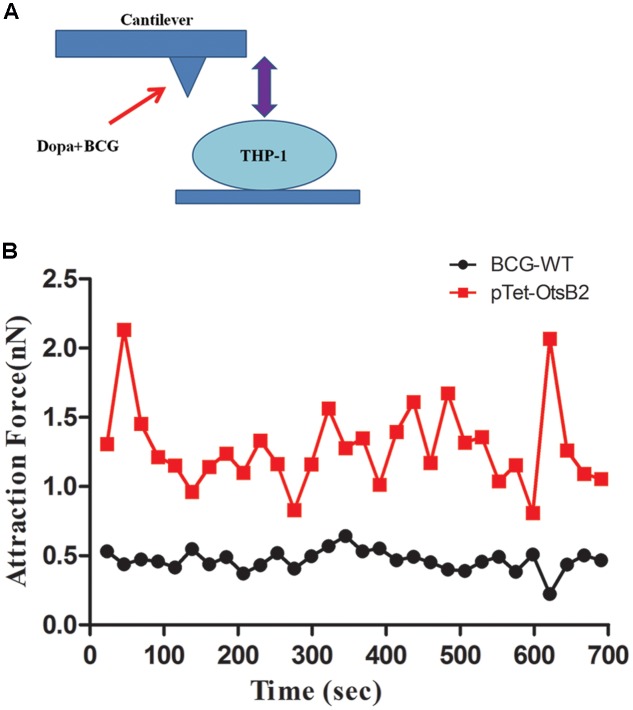
**BCG-OtsB2 has stronger binding interactions to THP-1 macrophages as measured by AFM.** **(A)** cartoon schematic of AFM measurements. **(B)** Binding force data for WT BCG (black) and BCG-OtsB2 (red) and THP-1 macrophages. Results representative of three experiments.

To further verify differences in binding of BCG-OtsB2 to THP-1 macrophages compared with WT BCG, we used atomic force microscopy to measure the binding force between the mycobacteria and the activated THP-1 macrophages. The greater the degree of binding, the greater the force required to separate the bacteria and host cells. As can be seen in **Figure 3** the binding force between BCG-OtsB2 and THP-1 cells was significantly greater than that between WT BCG and the macrophages, verifying that paradoxically, although phagocytosis of BCG-OtsB2 was decreased, this was in the context of greater binding to the macrophages.

## Discussion

Trehalose is present in most bacteria, fungi, plants and lower vertebrates, but not mammals, and has multiple roles in metabolism, as a structural precursor and as a stress response molecule. In mycobacteria, trehalose is a key component of a variety of glycolipids including TDM or cord factor, which is a key virulence factor (Kalscheuer and Koliwer-Brandl, 2014). Trehalose has been shown to be important for entry of *M. smegmatis* into stationary phase (Woodruff et al., 2004), and we found that over-expression of OtsB2 led to increased levels of trehalose and continued growth when WT BCG had entered stationary phase (**Supplementary Figure S1B**).

Bacterial inhibition of phagocytosis is well-described in pathogens that are usually described as extra-cellular (Ernst, 2000), but less commonly studied in bacteria that reside within an intra-cellular niche. Reports of mycobacterial capsular components (Stokes et al., 2004) and surface-exposed glycolipids (Villeneuve et al., 2005) that inhibit phagocytosis of mycobacteria suggest that although the macrophage is viewed as the cellular niche for pathogenic mycobacteria, the interplay between host-cell and pathogen may be subtle and complex. Stokes et al. (2004) showed that disruption of the carbohydrate-rich capsule of pathogenic mycobacteria led to increased phagocytosis, suggesting that WT mycobacteria are relatively anti-phagocytic. In contra-distinction to our findings, however, in their study, capsule-less mycobacteria showed both increased adhesion (at 4°C) and phagocytosis (37°C), whereas we found that BCG-OtsB2 showed a paradoxical relationship between increased adhesion – as measured both by increased attachment at 4°C (**Figure 2** and **Supplementary Figure S5**) and increased force required to disrupt association by AFM (**Figure 3**). It’s not clear how over-expression of OtsB2 would result in increased attachment but decreased phagocytosis. Using AFM, it had previously been shown that subtle differences in capsular composition of *E. coli* cells could alter the force of bacterial adhesion (Razatos et al., 1998). One possibility might be that over-expression of OtsB2 results in synthesis of trehalose-containing glycolipids or glycoproteins that specifically engage a macrophage receptor that is not competent for initiation of phagocytosis, and thereby limit engagement of phagocytic receptors. However, given the requirement for OtsA as well as OtsB activity for trehalose production, it is not clear if this occurred within our experimental system and changes to the components of the cell wall and capsule would need to be experimentally verified. We measured changes in expression of a number of known mycobacterial receptors expressed on macrophages, and did not find any changes when macrophages were infected by BCG-OtsB2 compared with WT BCG (**Supplementary Figure S3**). Given the large number and redundant nature of mycobacterial receptors (Ernst, 1998), we cannot exclude that expression of some other phagocytic receptors were altered. Non-phagocytic macrophage receptors are relatively less studied than their phagocytic counterparts (Gordon, 1995) but it is known that macrophages interact non-phagocytically with the glycan-rich components of the extra-cellular matrix (Gordon, 2003). Over-expression of OtsB2 may alter the surface components of BCG, which may alter its relative affinity for non-phagocytic receptors compared with WT bacteria.

## Conclusion

We have shown that over-expression of the Mycobacterial trehalose-phosphate phosphatase OtsB2 increases adhesion between BCG and activated THP-1 cells, whilst decreasing phagocytosis. These results highlight that phagocytosis of pathogen cells by macrophages is likely a subtle balance between benefit to host or pathogen, which may shift with minor changes in either bacterial or host cell heterogeneity (Cambier et al., 2014). Further research into the mechanisms of host–pathogen interactions will aid the understanding of host immunity to mycobacterial infections and potentially the rational design of more effective vaccines

## Author Contributions

HL designed and performed research and analyzed results. MW and YS assisted with AFM experiments and analysis of those data. BJ oversaw the project, designed research and analyzed data. HL and BJ wrote the paper with input from other authors.

## Conflict of Interest Statement

The authors declare that the research was conducted in the absence of any commercial or financial relationships that could be construed as a potential conflict of interest.

## Supplementary Material

**FIGURE S1 | OtsB2 over-expression increases bacteria growth in the stationary phase.** **(A)** Western Blot of WT BCG and BCG-OtsB2 following induction with 50 ng/ml ATc probed by primary antibody against hexahistidine tag, which is N-terminally expressed in the recombinant OtsB2 protein. Lanes (1) WT BCG log phase, (2) WT BCG early stationary phase, (3) WT BCG mid-stationary phase, (4) BCG-OtsB2 log phase, (5) BCG-OtsB2 early stationary phase, (6) BCG-OtsB2 mid-stationary phase **(B)** BCG over-expressing OtsB2 (pTet-OtsB2) grew to denser culture during stationary phase. **(C)** Late stationary phase was associated with increased cellular content of trehalose in pTet-OtsB2 cells. Results representative of at least two different experiments. ^∗^*p* < 0.05, ^∗∗∗^*p* < 0.001 by Student’s *t*-test.

**FIGURE S2 | BCG-OtsB2 infection causes upregulation of CD1d surface expression.** Analysis of surface marker staining of WT BCG and BCG-OtsB2 infected THP-1 macrophages was analyzed by flow cytometry. ^∗^*p* < 0.05, ^∗∗^*p* < 0.01, ^∗∗∗^*p* < 0.001 by Student’s *t*-test. Results representative of two experiments.

**FIGURE S3 | BCG-OtsB2 infection does not change expression of mycobacterial phagocytosis receptors on THP-1 macrophages compared with WT BCG.** Analysis of surface marker staining of WT BCG and BCG-OtsB2 infected THP-1 macrophages was analyzed by flow cytometry. ^∗^*p* < 0.05 by Student’s *t*-test. Results representative of two experiments.

**FIGURE S4 | THP-1 macrophages infected by OtsB2 do not have a general phagocytosis defect.** **(A)** Flow cytometry of macrophages alone (no beads). **(B)** Flow data of macrophages fed fluorescent beads only. Flow data of macrophages fed fluorescent beads following infection by WT BCG **(C)** and BCG-OtsB2 **(D)**. Results representative of three separate experiments.

**FIGURE S5 | BCG-OtsB2 has greater binding but decreased phagocytosis by RAW264.7 macrophages than WT BCG.** Phagocytosis analysis by flow cytometry of WT BCG and BCG-OtsB2 by RAW264.7 macrophages following 24 h incubation at 37°C [representative flow cytometry data in **(A)** and quantification of biological triplicates in **(B)**]. Binding analysis of BCG-OtsB2 and BCG to RAW264.7 macrophages at 4°C for 4 h [representative flow cytometry data in **(C)** and quantitation of biological triplicates in **(D)**]. ^∗^*p* < 0.05, ^∗∗∗^*p* < 0.001 by Student’s *t*-test. Results representative of at least two separate experiments.
