# Supplementary figures and images for: Over-Expression of the Mycobacterial Trehalose-Phosphate Phosphatase OtsB2 Results in a Defect in Macrophage Phagocytosis Associated with Increased Mycobacterial-Macrophage Adhesion
Source: Front Microbiol. 2016 Nov 4;7:1754. doi: 10.3389/fmicb.2016.01754 (PMC5095139; doi:10.3389/fmicb.2016.01754)

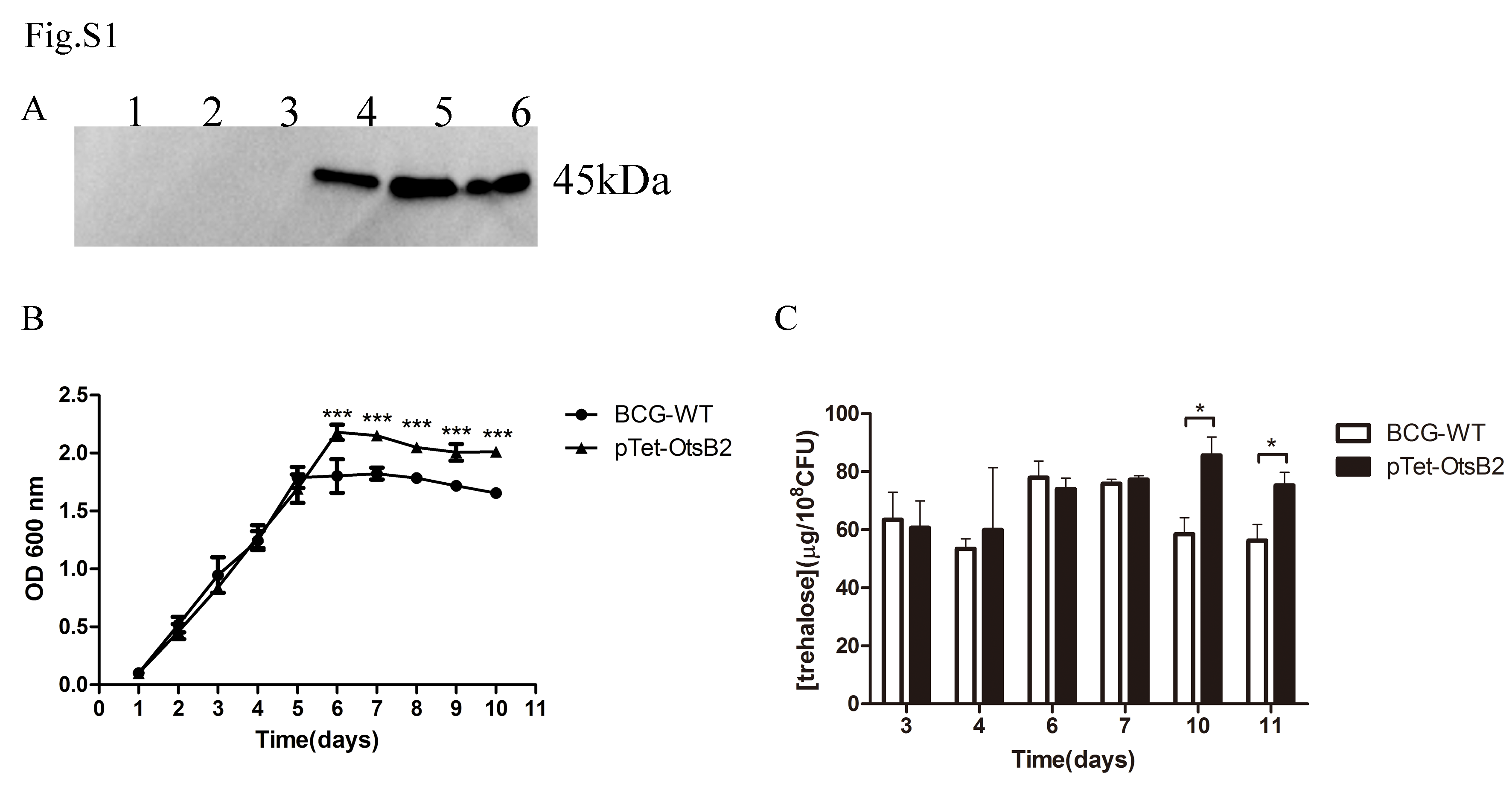

Supplement: Supplementary file 1 [file Image_1.TIF]

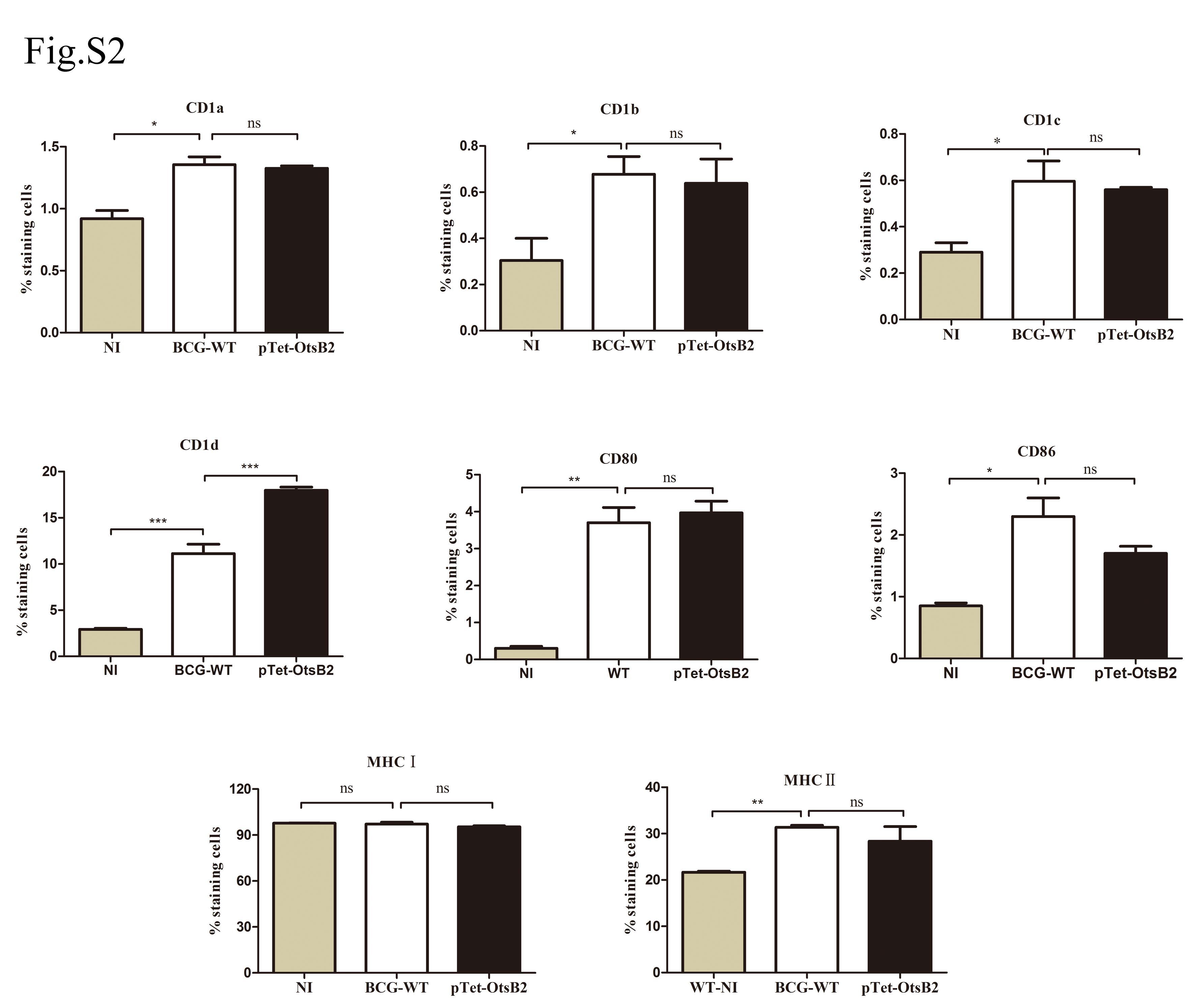

Supplement: Supplementary file 2 [file Image_2.TIF]

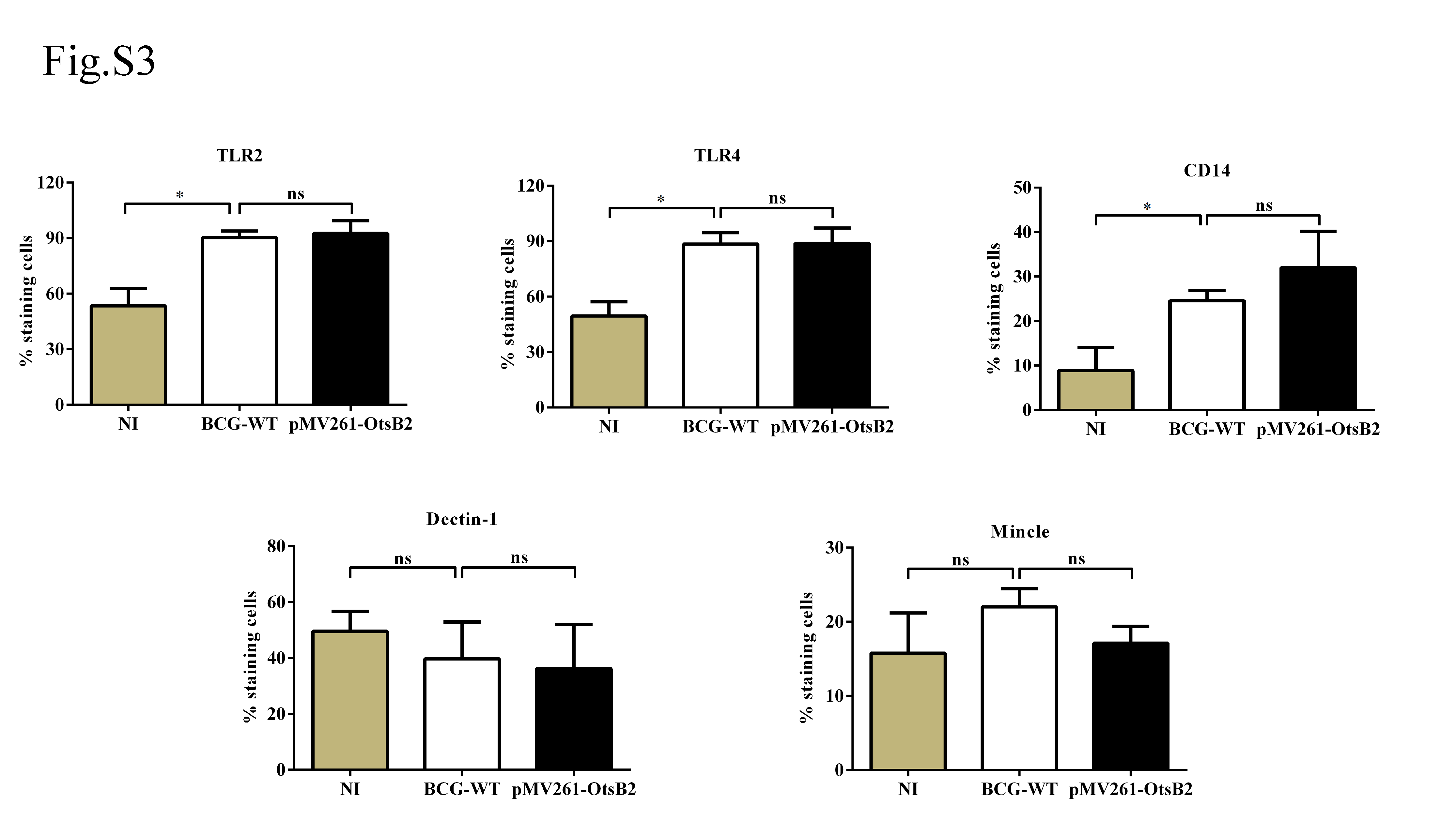

Supplement: Supplementary file 3 [file Image_3.TIF]

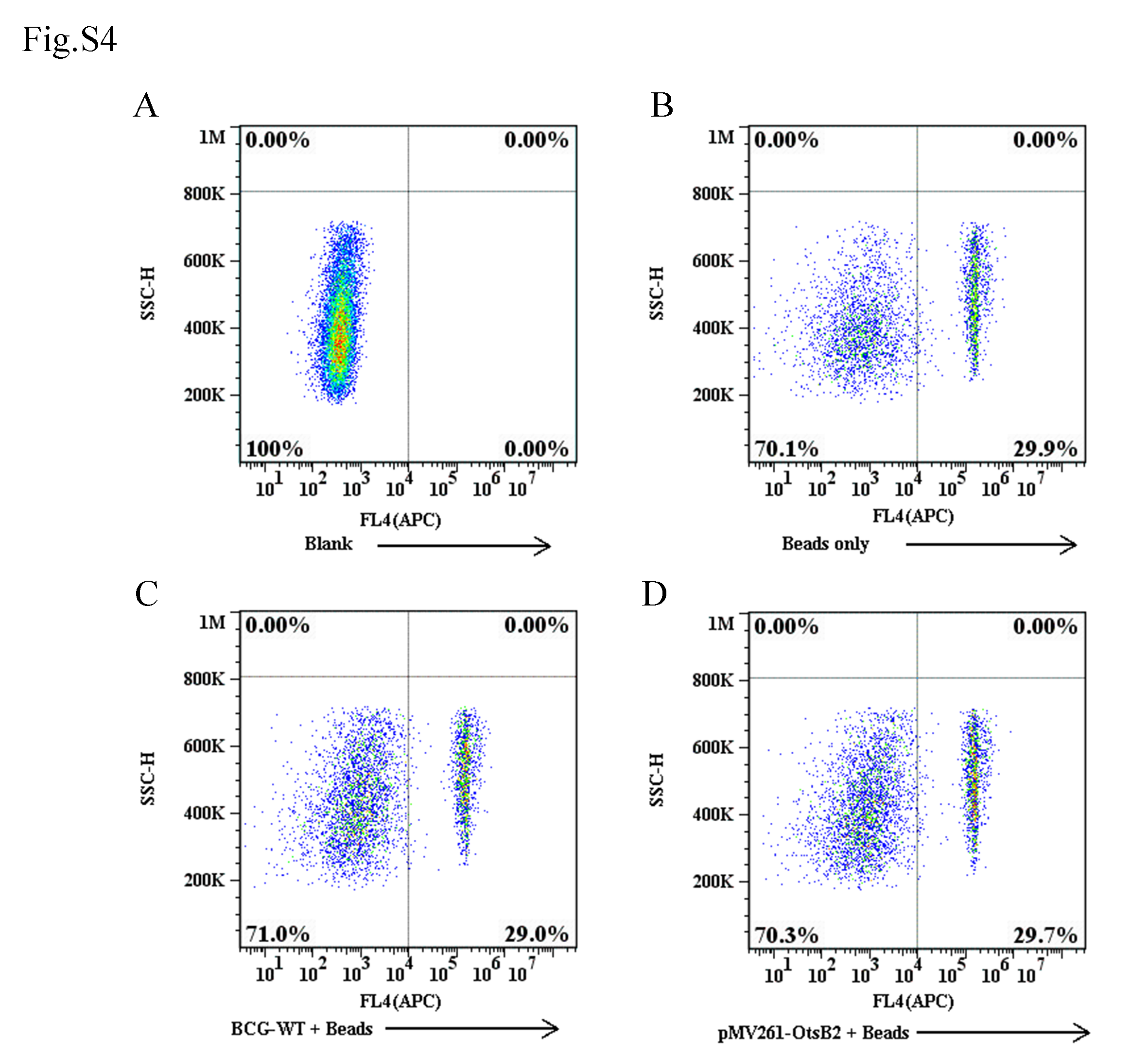

Supplement: Supplementary file 4 [file Image_4.TIF]

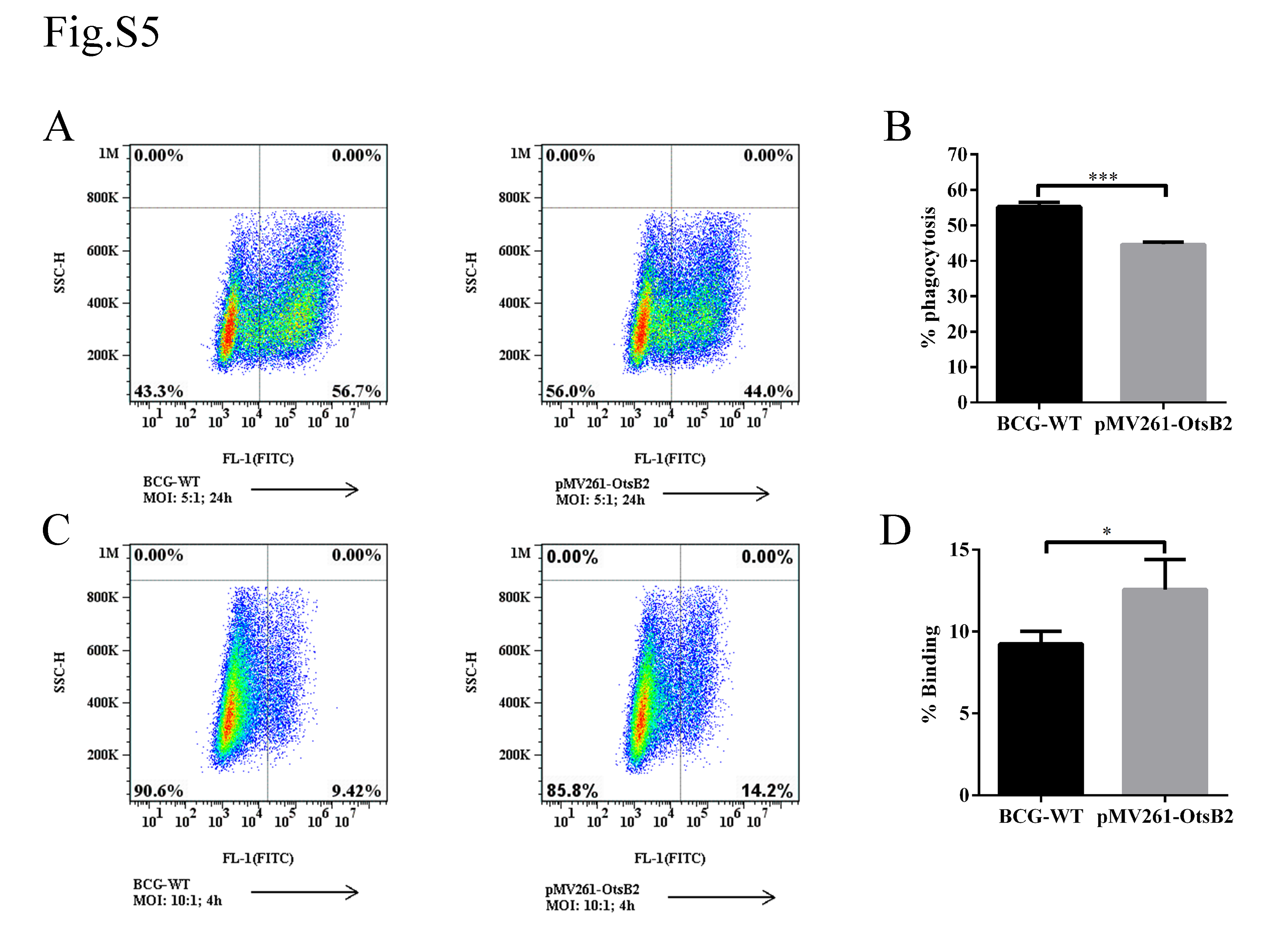

Supplement: Supplementary file 5 [file Image_5.TIF]
